# A Role for Syntaxin 3 in the Secretion of IL-6 from Dendritic Cells Following Activation of Toll-Like Receptors

**DOI:** 10.3389/fimmu.2014.00691

**Published:** 2015-01-26

**Authors:** Laura E. Collins, Joseph DeCourcey, Keith D. Rochfort, Maja Kristek, Christine E. Loscher

**Affiliations:** ^1^Immunomodulation Research Group, School of Biotechnology, Dublin City University, Dublin, Ireland; ^2^School of Biotechnology, Dublin City University, Dublin, Ireland

**Keywords:** dendritic cells, SNAREs, STX3, IL-6, MIP-1α

## Abstract

The role of dendritic cells (DCs) in directing the immune response is due in part to their capacity to produce a range of cytokines. Importantly, DCs are a source of cytokines, which can promote T cell survival and T helper cell differentiation. While it has become evident that soluble-N-ethylmaleimide-sensitive-factor accessory-protein receptors (SNAREs) are involved in membrane fusion and ultimately cytokine release, little is known about which members of this family facilitate the secretion of specific cytokines from DCs. We profiled mRNA of 18 SNARE proteins in DCs in response to activation with a panel of three Toll-like receptors (TLR) ligands and show differential expression of SNAREs in response to their stimulus and subsequent secretion patterns. Of interest, STX3 mRNA was up-regulated in response to TLR4 and TLR7 activation but not TLR2 activation. This correlated with secretion of IL-6 and MIP-1α. Abolishment of STX3 from DCs by RNAi resulted in the attenuation of IL-6 levels and to some extent MIP-1α levels. Analysis of subcellular location of STX3 by confocal microscopy showed translocation of STX3 to the cell membrane only in DCs secreting IL-6 or MIP-1α, indicating a role for STX3 in trafficking of these immune mediators. Given the role of IL-6 in Th17 differentiation, these findings suggest the potential of STX3 as therapeutic target in inflammatory disease.

## Introduction

Since the discovery of soluble-N-ethylmaleimide-sensitive-factor accessory-protein receptors (SNAREs) in the 1980s they have been defined to have an essential role for the trafficking of molecules and membranes within cells (1). To date, there are 38 members of the SNARE family and the subcellular locations and differential combinations of SNARE proteins differ between cell types. The pairing of SNARE proteins is selective, which limits trafficking and membrane fusion between intracellular organelles or membrane fusion (2). Studies investigating the expression of individual SNAREs and their subcellular locations have led to the mapping of intracellular pathways. More advanced work has begun to assign functions to SNAREs in specific cellular immune responses such as the role of STX6 and Vti1b in the secretion of TNF from activated macrophage (3).

Dendritic cells are essential for the generation of a functional immune response. They do so by the capture and processing of antigens, expression of surface co-stimulatory molecules, migration to lymphoid organs, and secretion of cytokines and chemokines (4). Understanding these actions, such as their ability to drive T helper cell responses makes the dendritic cells (DC) a powerful tool for manipulating the immune system. Furthermore, the cytokines they secrete are associated with the pathogenesis of a wide range of inflammatory diseases (5). For example, blockade of TNF-α has been clinically successful in a number of immune mediated pathologies such as rheumatoid arthritis, Crohn’s disease, and psoriasis (6). Abolishment of IL-23 by either utilizing IL-23 knockout mouse models or subjecting wild-type mice to anti-IL-23 treatment has been shown to render mice resistant to certain inflammatory and autoimmune diseases such as experimental autoimmune encephalitis (EAE), collagen-induced arthritis (CIA), and inflammatory bowel disease (IBD) (7–9). As DCs act as a link between innate and adaptive immunity and secrete cytokines, which are important in inflammatory diseases, investigating how DCs secrete these cytokines may provide us with new therapeutic targets in these diseases.

To date, there has been little work exploring the expression or function of SNAREs in DCs. The most notable studies include a profile of SNARE expression in DCs of Vti1a, Vti1b, VAMP3, VAMP8 and STX8, which were up-regulated in response to acetylsalicylic acid (ASA) (10). Furthermore, Ho et al., in 2008 attributed a functional role to VAMP8 in DCs. They demonstrated that over-expression of VAMP8 resulted in significant inhibition of phagocytosis and VAMP8^−/−^ DCs had significantly increased phagocytic ability indicating that VAMP8 could inhibit phagocytosis from DCs (11).

To investigate the role of SNAREs in DC secretion we profiled the expression of mRNA following activation with a panel of Toll-like receptors (TLR) ligands using the established JAWS II DC cell line. We hypothesized that an up-regulation of particular SNAREs in DCs following activation may be indicative of a role for them in the secretion of cytokines or chemokines. This is the first study to demonstrate that activation of DCs with TLR ligands results in the differential expression of SNARE proteins, which correlates with the profile of cytokines and chemokines being secreted by the cell. Furthermore, we confirmed a role for STX3 in the secretion of IL-6 using RNAi. This study suggests that SNARE proteins may provide new therapeutic targets in inflammatory disease.

## Materials and Methods

### Animals and materials

C57BL/6 mice were purchased from Charles River at 6–8 weeks of age. Animals were housed in a licensed BioResource Unit (Dublin City University), according to Health Products Regulatory Authority (HPRA) regulations and had *ad libitum* access to animal chow and water. LPS *E. coli* serotype R515 was purchased from Enzo Life Sciences. Loxoribine and PGN from InvivoGen, rGMCSF from Sigma Aldrich, and STX3 siRNA from Life Technologies.

### Dendritic cell culture

A murine DC line JAWS II (CRL-11904) was purchased from ATCC and maintained in fully supplemented α-MEM supplemented with 10% (v/v) fetal bovine serum (FBS), 2% (v/v) Penicillin–Streptomycin, and 5 ng/ml rGM-CSF. Bone marrow dendritic cells (BMDCs) were harvested from the femurs and tibia of mice and were also cultured in fully supplemented α-MEM. On day three, supernatants were removed from BMDCs and replaced with fresh fully supplemented α-MEM. On day seven BMDCs were used for subsequent experiments.

### Quantitative polymerase chain reaction

JAWS II DCs were cultured for 7 days in the presence of murine recombinant GM-CSF (Sigma Aldrich) and plated at 1 × 10^6^ cell/ml. Cells were then stimulated with 5 μg/ml PGN, 100 ng/ml LPS (*E. coli* serotype R515), or 1 mM Loxoribine over a time-course. Total RNA was extracted, converted to cDNA, which was then subjected to quantitative real-time PCR. 2^−ΔΔCq^ formula of the delta–delta Cq method was used and a fold change in expression of SNARE genes was normalized against that of endogenous control S18. SNARE mRNA levels were determined relative to expression in the absence of TLR stimulation, which is given an arbitrary value of 1. All primer sequences are listed in Table 1.

**Table 1:** **Primers used for PCR and their sequences**.

Gene	Ref Seq ID	Forward [5′–3′]	Reverse [5′–3′]
SNAP-23	NM_009222	GTTCTTGCTCAGGCTTCC	CCAACCAACCAATACCAATAATG
STX-2	NM_007941	GGTGGCAAAGGTGATGTT	CAGGTATGGTCGGAGTCA
STX-3	NM_001025307	CCACAACCACTAGCATCATAA	CTCAAGAGATATTCCGCCTTAA
STX-4	NM_009294	GGTGTCAAGTGTGAGAGAG	AACCTCATCTTCATCGTCTG
STX-5	NM_001167799.1	GCAAGTCCCTCTTTGATGAT	TTCAGATTCTCAGTCCTCACT
STX-6	NM_021433	CAAGGATTGTTTCAGAGATGGA	CCTGACAATTTGCCGAGTA
STX-7	NM_016797	CACAACGCATCTCCTCTAAC	TAATCGGTCTTTCTGTATCTTTCTC
STX-11	NM_001163591.1	ATCACGGCAAAATGAAGGA	GGTCGGTCTCGAACACTA
STX-12	NM_133887	CGCAAGAAGATGTGTATCCT	CTCTGAGGCAAGCACTTC
STX-16	NM_001102432.1	GAGCAGTACCAGAAGAAGAAC	CAAGTCCTATCACCAATAATCCA
Vti1a	NM_016862	GAATGTATAGCAACAGGATGAGA	CCGTGTTATCCAGCAGATG
Vti1b	NM_016862	TACCTTGGAGAACGAGCAT	TGGACATTGAGCGAAGAATC
VAMP-1	NM_001080557	CCCTCTGTTTGCTTTCTCA	CGTTGTCTTCGGGTAGTG
VAMP-2	NM_009497	CTCCTTCCCTTGGATTTAACC	TGAAACAGACAGCGTATGC
VAMP-3	NM_009498	TTGTTCTTGTTGTATATCACTCCTAA	GGCTCGCTCTCACAGTAT
VAMP-4	NM_016796	GTATGCCTCCCAAGTTCAAG	TGTAGTTCATCCAGCCTCTC
VAMP-7	NM_011515	GATGGAGACTCAAGCACAAG	GACACAATGATATAGATGAACACAAT
VAMP-8	NM_016794	GGCGAAGTTCTGCTTTGA	CTTGACTCCCTCCACCTC
S18	NM_011296	CTGAGAAGTTCCAGCACATT	GCTTTCCTCAACACCACAT

### DC stimulation and measurement of cytokines and chemokines

JAWS II DCs or BMDCs were stimulated with LPS (100 ng/ml), Loxoribine (1 mM) or PGN (5 μg/ml) at timepoints between 0 and 12 h. Culture supernatants were removed and stored at −80°C until further analysis. IL-1β, IL-6, TNFα, MIP-1α concentrations in cell culture supernatants were analyzed by DuoSet ELISA Kits (R&D Systems) according to manufacturer’s instructions.

### RNAi of STX3

JAWS II DCs were plated and left to rest overnight. GeneSilencer^®^ (Genelantis) reagent and serum free media were added to tube one. siRNA diluent, specific STX3 siRNAs (Invitrogen™) (1 nM) (Table 2) or 1 nM scrambled non-silencing siRNA or 1 nM of fluorescently labeled Cy™ 3 labeled glyceraldehyde 3-phosphate dehydrogenase (GAPDH) (Invitrogen™) and serum free media were added to tube two and left to incubate at RT for 5 min. The two tubes were then mixed together and incubated for 10 min. This mixture was then added directly to the plate and left for 24 h. Medium was then removed, replenished with fresh media with and without LPS 100 ng/ml and left for another four hours. Supernatants were removed and analyzed for the levels of IL-1β, IL-6, TNF-α, and MIP-1α. Western blot was performed to verify STX3 knockdown.

**Table 2:** **STX3 siRNA sequences**.

Sequence (5′–3′)	Sense	Antisense
STX3 #1	CAGCCUUCAUGGACGAGUUtt	AACUCGUCCAUGAAGGCUGtg
STX3 #2	GGAAGAUGAGGUUCGGUCAtt	UGACCGAACCUCAUCUUCCtc

### Confocal microscopy

0.13–0.16 mm thick coverslips were placed in a six-well plate. BMDCs harvested from C57BL/6 mice and JAWS II DCs were added and left over-night to adhere. Cells were then stimulated with LPS (100 ng/ml), Loxoribine (1 mM), and PGN (5 μg/ml) for 1 h. Cells were washed and fixed with 3% Paraformaldehyde (PAF) and this reaction was then quenched with 50 mM Ammonium Chloride. Cells were permeabilized with 0.1% saponin, 0.25% fish gelatin and 0.02% sodium azide in PBS. STX3 primary antibody was added to the coverslips and incubated overnight at 4°C. Corresponding fluorescently conjugated secondary antibody (AlexaFluro 488 or AlexaFluro 546) was then added to the coverslips and incubated for 1 h at room temperature. Nuclei were stained with propidium iodide dye (PI) and samples mounted using DAKO anti-fade medium. Slides were then viewed using a Zeiss 710 confocal microscope and analyzed using LSM software.

### Statistics

The parametric student’s *t*-test was used using Excel (Microsoft) to determine significant differences between two samples. The level of statistical significance was indicated by *p ≤ 0.05, **p ≤ 0.01, ***p ≤ 0.001.

## Results

### SNARE mRNA expression in DCs following activation with a panel of TLR ligands

**Figure 1:**
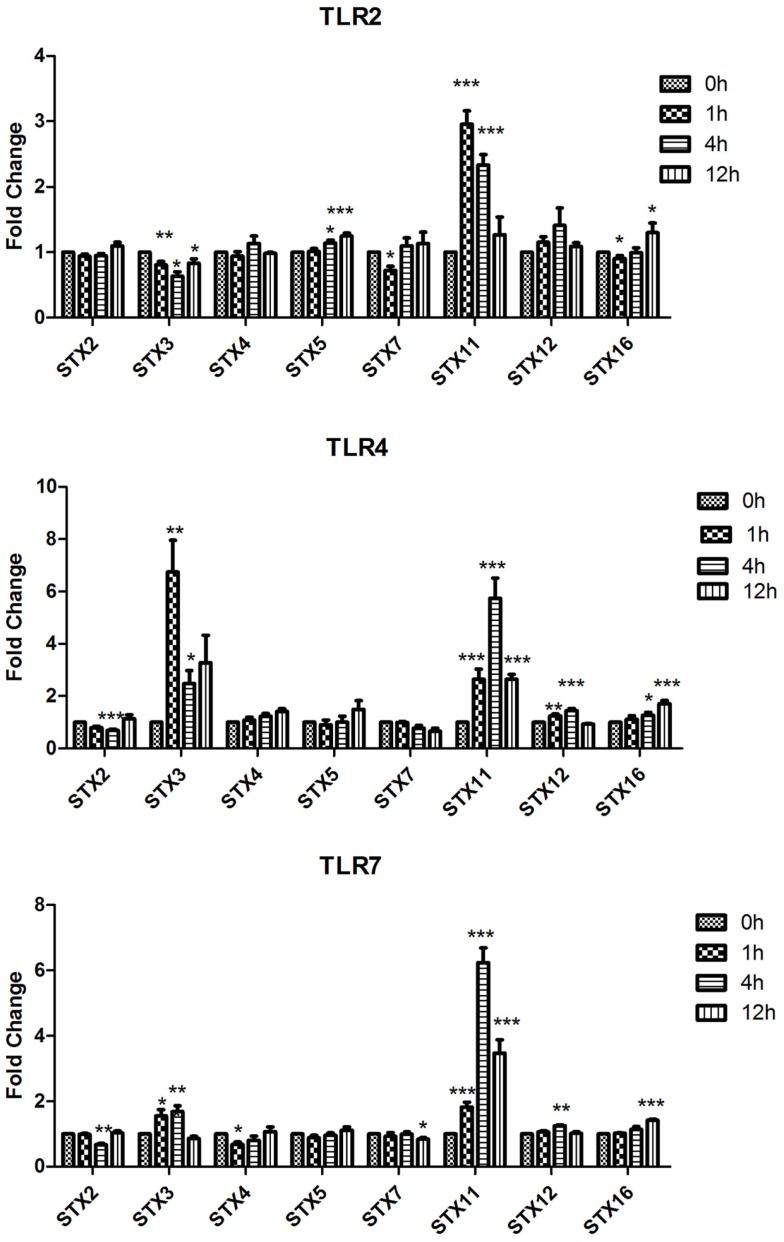
**Effect of TLR ligand stimulation on Qa SNAREs (STX2, STX3, STX4, STX5, STX7, STX11, STX12, and STX16) mRNA expression in JAWS II DCs**. JAWS II DCs were plated 1 × 10^6^/ml and stimulated with 100 ng/ml LPS, 1 mM Loxoribine, or 5 μg/ml PGN at 1, 4, and 12 h. The amount of specific SNARE protein was quantified by reverse transcription followed by RT-qPCR and normalized with S18 levels. Fold differences were calculated relative to SNARE levels at time zero (assigned value of 1). Results are mean ± SEM of quadruplicate assays. A two sample, two-tailed student’s *t*-test comparing ΔCts of control and 1, 4, or 12 h stimulated sample *p ≤ 0.05, **p ≤ 0.01, ***p ≤ 0.001

**Figure 2:**
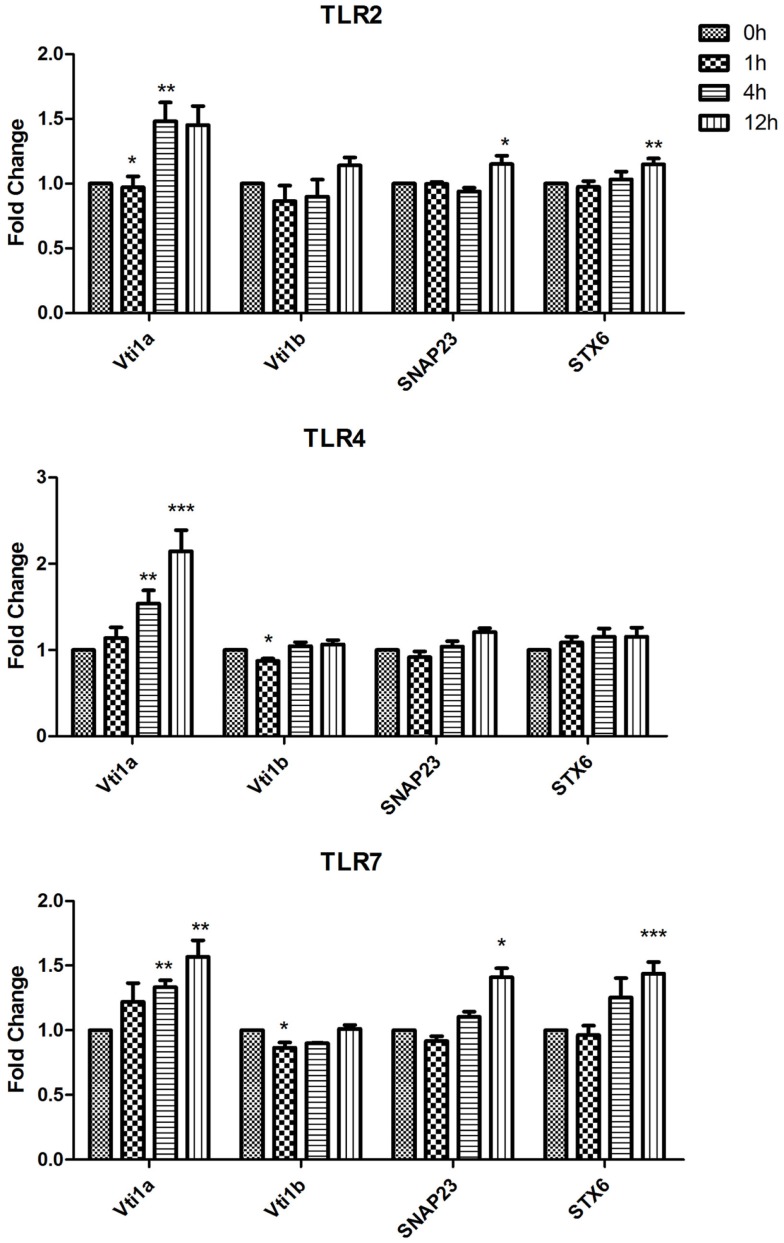
**Effect of TLR ligand stimulation on Qb SNARES (Vti1a and Vti1b), Qbc SNARE (SNAP23), Qc SNARE (STX6) mRNA expression in JAWS II DCs**. JAWS II DCs were plated 1 × 10^6^/ml and stimulated with 100 ng/ml LPS, 1 mM Loxoribine, or 5 μg/ml PGN at 1, 4, and 12 h. The amount of specific SNARE protein was quantified by reverse transcription followed by RT-qPCR and normalized with S18 levels. Fold differences were calculated relative to SNARE levels at time zero (assigned value of 1). Results are mean ± SEM of quadruplicate assays. A two sample, two tailed student’s *t*-test comparing ΔCts of control and 1, 4, or 12 h stimulated sample *p ≤ 0.05, **p ≤ 0.01, ***p ≤ 0.001.

**Figure 3:**
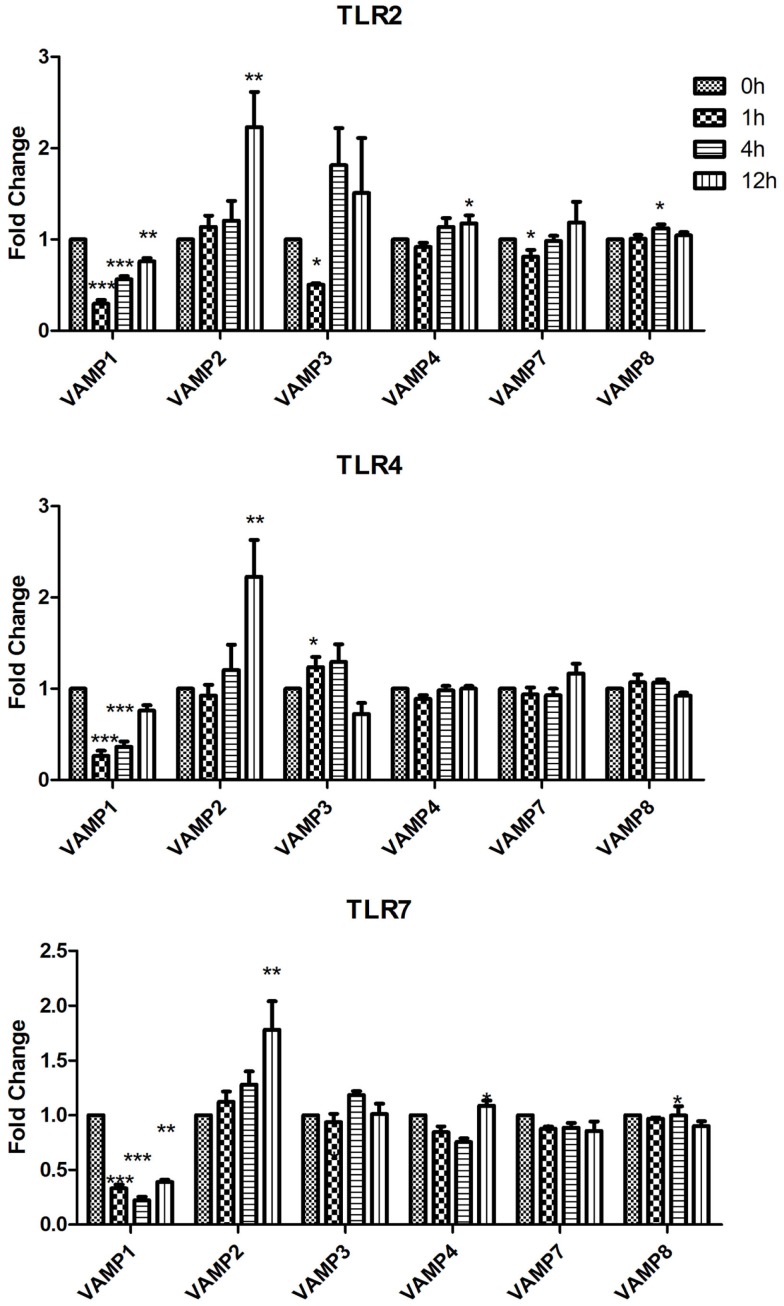
**Effect of TLR ligand stimulation on R SNAREs (VAMP1, VAMP2, VAMP3, VAMP4, VAMP7, and VAMP8) mRNA expression in JAWS II DCs**. JAWS II DCs were plated 1 × 10^6^/ml and stimulated with 100 ng/ml LPS, 1 mM Loxoribine, or 5 μg/ml PGN at 1, 4, and 12 h. The amount of specific SNARE protein was quantified by reverse transcription followed by RT-qPCR and normalized with S18 levels. Fold differences were calculated relative to SNARE levels at time zero (assigned value of 1). Results are mean ± SEM of quadruplicate assays. A two sample, two tailed student’s *t*-test comparing ΔCts of control and 1, 4, or 12 h stimulated sample *p ≤ 0.05, **p ≤ 0.01, ***p ≤ 0.001.

Using RT-quantitative polymerase chain reaction (qPCR) we analyzed the expression of Qa SNAREs (STX2, STX3, STX4, STX5, STX7, STX11, STX12, and STX16) (Figure 1), Qb SNARES (Vti1a and Vti1b), Qbc SNARE (SNAP23), Qc SNARE (STX6)(Figure 2), and R SNAREs (VAMP1, VAMP2, VAMP3, VAMP4, VAMP7, and VAMP8)(Figure 3) in JAWS II DCs following stimulation with 100 ng/ml LPS, 1 mM Loxoribine, or 5 μg/ml PGN. RT-qPCR was normalized with S18 levels. This housekeeping gene showed smallest standard deviation compared to β-actin and GAPDH between the technical replicates and LPS, Loxoribine and PGN stimulation. Thus was used for all subsequent experiments (Figure S1 in Supplementary Material).

The most significant fold change increases in mRNA expression in the Qa SNAREs over time were that of STX3 and STX11 (Figure 1). Expression of STX3 mRNA in JAWS II DCs significantly increased following activation with LPS and Loxoribine post 1 and 4 h stimulation and conversely was significantly down-regulated following PGN activation (*p* ≤ 0.01 and *p* ≤ 0.05). STX11 mRNA expression in JAWS II DCs increased significantly following activation with LPS, Loxoribine and PGN at 1 and 4 h compared to control cells (*p* ≤ 0.001) (Figure 1).

In the assessment of expression of Qb SNARES (Vti1a and Vti1b), Qbc SNARE (SNAP23), and Qc SNARE (STX6) there was a significant up-regulation of Vti1a mRNA at 4 and 12 h post LPS and Loxoribine stimulation (*p* ≤ 0.01, *p* ≤ 0.001, *p* ≤ 0.01) in comparison to control cells (Figure 2).

While VAMP1 was significantly down-regulated following stimulation with LPS, Loxoribine, and PGN; VAMP2 was significantly increased at 12 h post stimulation with these ligands (Figure 3). Following Loxoribine stimulation VAMP8 expression was significantly down-regulated at 12 h (*p* ≤ 0.05) and up-regulated following PGN stimulation after 4 h. LPS stimulation did not have any significant effect on VAMP8 expression (Figure 3).

Our data demonstrate that SNAREs are regulated in JAWS II DCs in response to TLR ligation and that their expression differed depending on the type of TLR ligand used to activate the cell (Figures 1–3).

### Secretion of cytokines and chemokines by DCs following activation with a panel of TLR ligands

**Figure 4:**
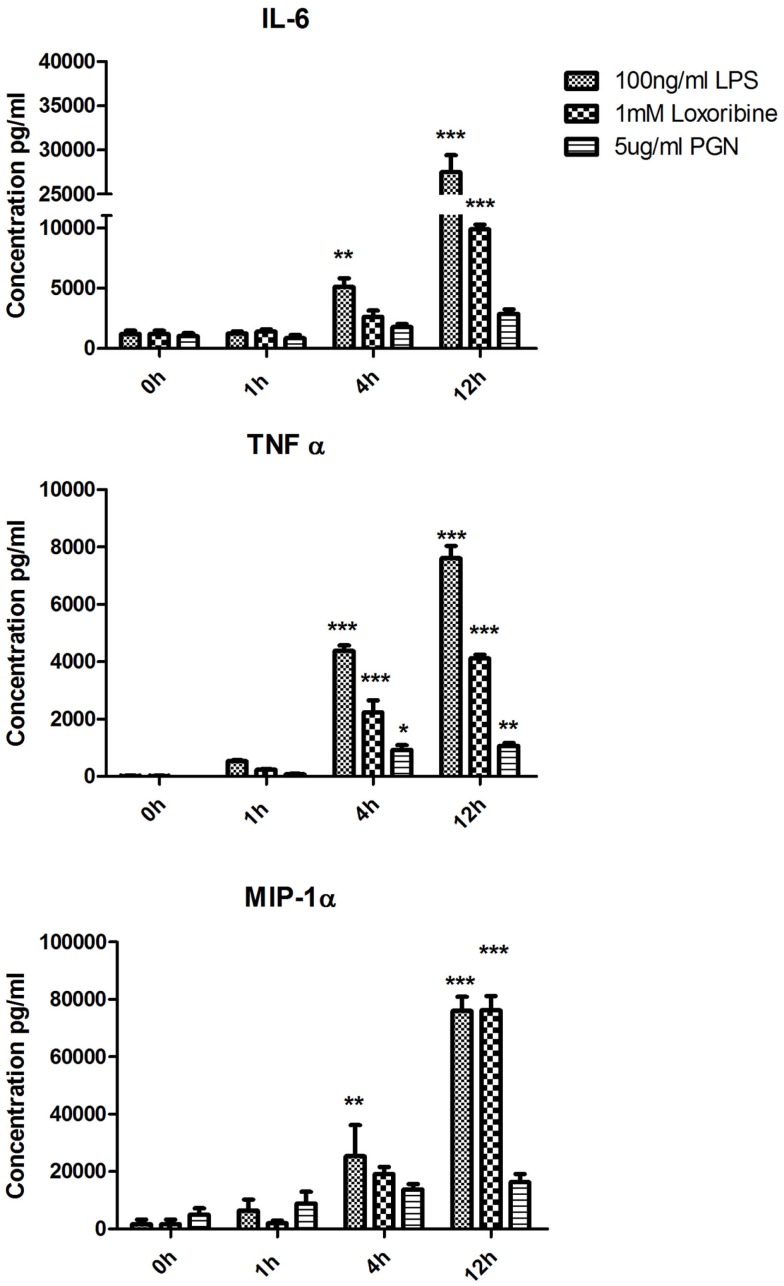
**Time-course of cytokine and chemokine secretion from JAWS II DCs in response to TLR4, TLR7, and TLR2 activation**. JAWS II DCs were cultured in r-GM-CSF for 7 days and subsequently were plated (1 × 10^6^ cells/ml) and left to rest overnight. Cells were then stimulated with 100 ng/ml LPS, 1 mM Loxoribine, and 5 μg/ml PGN. Supernatants were recovered after 1, 4, and 12 h and assessed for levels of cytokines using specific immunoassays. Results are ±SEM of quadruplicate assays and represent three independent experiments. *p ≤ 0.05, **p ≤ 0.01, and ***p ≤ 0.001 comparing control versus stimulated JAWS II DCs as determined by a two-tailed*t*-test.

To identify patterns in cytokine and chemokine secretion that may correlate with the expression of SNAREs, we analyzed the secretion of IL-6, TNF-α, and MIP-1α over the same time-course of 1, 4, and 12 h (Figure 4). IL-6 secretion was significantly up-regulated from JAWS II DCs following LPS stimulation at 4 h and 12 h and Loxoribine at 12 h (*p* ≤ 0.01, *p* ≤ 0.001) but remained unchanged following PGN stimulation. TNF-α secretion was significantly up-regulated at 4 and 12 h following LPS, Loxoribine, and PGN stimulation (*p* ≤ 0.001, *p* ≤ 0.05 and *p* ≤ 0.01) and MIP-1α secretion was significantly up-regulated following LPS stimulation at 4 and 12 h (*p* ≤ 0.01 and *p* ≤ 0.001) and Loxoribine stimulation at 12 h (*p* ≤ 0.001) but not with PGN stimulation (Figure 4). The trend of expression of IL-6 and TNF-α in response to LPS and Loxoribine stimulation at 4 and 12 h and MIP-1 α at 4 h post correlated with up-regulation of STX3 mRNA expression at 1 h following LPS and Loxoribine stimulation but not PGN stimulation (Figures 1 and 4).

### Knockdown of STX3 significantly decreases the secretion of IL-6 and MIP-1 alpha from JAWS II DCs

**Figure 5:**
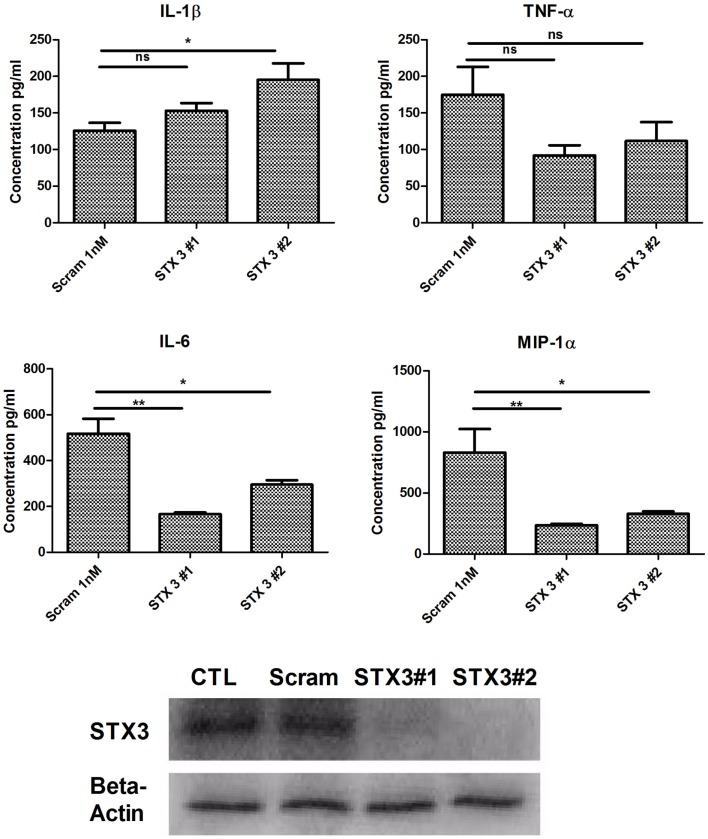
**Knockdown of STX3 by siRNA**. JAWS II DCs were transfected with siRNAs against STX3 (STX3 #1 and STX3 #2) or a negative control non-silencing siRNA. At 24 h after the transfection, new media was put on the cells and left for 4 h. Lysates and media were then harvested. Basal levels of cytokines or chemokines were assessed using specific immunoassays and knockdown was confirmed at protein level by western blot. *p < 0.05 and **p < 0.01 comparing control versus STX3 siRNA transfected JAWS II DCs as determined by a two-tailed *t*-test.

As previous studies have indicated a role for STX3 in secretion of chemokines (12) and we have demonstrated that increased expression of STX3 correlated with cytokine and chemokine secretion we examined whether abolishing STX3 expression would have any effect on cytokine or chemokine secretion from DCs. STX3 was knocked down using STX3 specific siRNAs (Invitrogen™). Transfection efficiency was confirmed with Cy3 GAPDH (Figure S2 in Supplementary Material) and STX3 knockdown by both siRNAs was confirmed at the protein level by using Western blot (Figure 5). Supernatants were subsequently removed 4 h later and analyzed for basal levels of IL-1β, IL-6, TNF-α, and MIP-1α (Figure 5). In the absence of STX3, secretion of IL-6 and MIP-1α were significantly decreased (*p* ≤ 0.05 and *p* ≤ 0.01) whereas IL-1β was significantly increased (*p* ≤ 0.05), with no significant changes in TNF-α secretion (Figure 5).

### STX3 translocates to the plasma membrane only in IL-6 secreting JAWS II DCs and BMDCs

**Figure 6:**
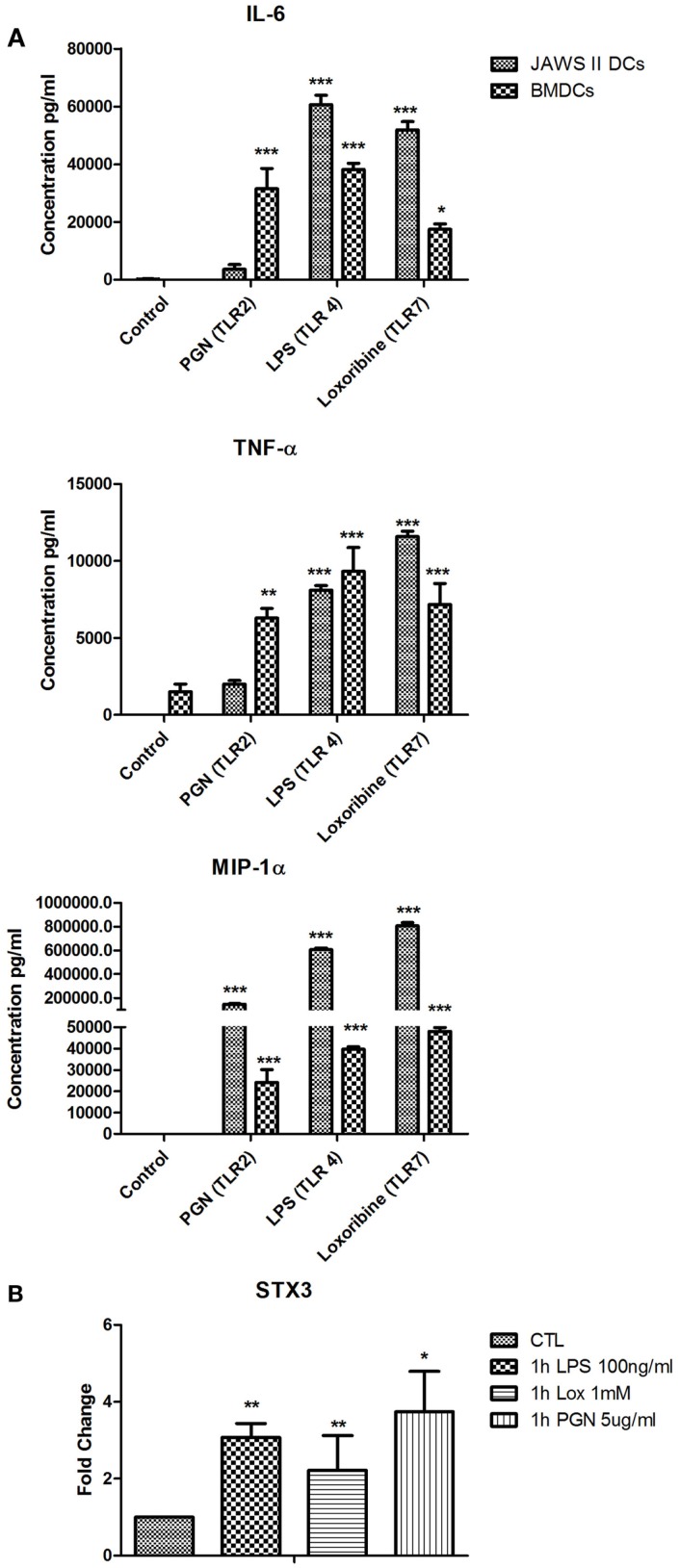
**(A)** Cytokine and chemokine secretion from BMDC versus JAWS II DCs in response to TLR2, TLR4, and TLR7. **(B)** Effect of TLR ligand stimulation on Qa SNARE, STX3 mRNA expression in BMDCs. DCs were cultured in r-GM-CSF for 7 days and subsequently were plated (1 × 10^6^ cells/ml) and left to rest overnight. Cells were then stimulated with 100 ng/ml LPS, 1 mM Loxoribine, and 5 μg/ml PGN and mRNA and supernatants were recovered after 1 or 24 h Supernatants were assessed for levels of cytokines using specific immunoassays and the amount of STX3 was quantified by reverse transcription followed by RT-qPCR and normalized with S18 levels. Fold differences were calculated relative to SNARE levels at time zero (assigned value of 1). Results are ±SEM of quadruplicate assays and represent three independent experiments. *p ≤ 0.05, **p ≤ 0.01, and ***p ≤ 0.001 comparing control versus stimulated JAWS II DCs or BMDCs as determined by a two-tailed*t*-test.

We next used primary BMDCs to confirm our findings from the JAWS II DC line. We analyzed secretion of IL-6, TNF-α, and MIP-1α from BMDCs activated with LPS, Loxoribine, and PGN and demonstrated that IL-6 is significantly secreted from BMDCs when activated with all three TLR ligands (Figure 6A). mRNA of STX3 was then examined in BMDCs and was up-regulated in all instances where TLR activation of BMDCs resulted in IL-6 secretion, which supports our findings in the JAWS II cell line (Figure 6B).

**Figure 7:**
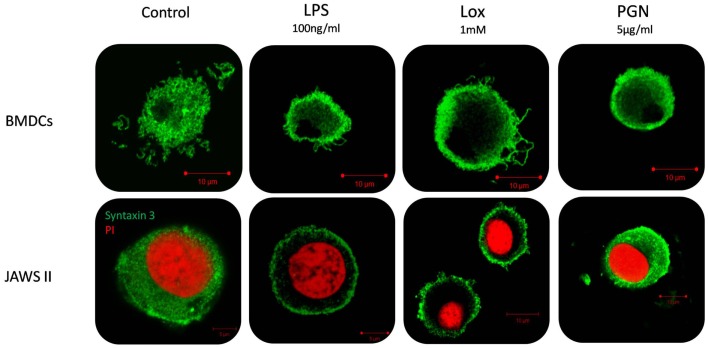
**Localization of STX3 to the plasma membrane in JAWS II DCs and BMDCs**. JAWS II DCs and BMDCs were plated 1 × 10^6^/ml and stimulated with 5 μg/ml PGN, 100 ng/ml LPS, and 1 mM Loxoribine for 1 h. Immunofluorescent indirect double staining of STX3 (Green) and nuclei (red) in TLR4, TLR7, and TLR2 stimulated JAWS II DCs and BMDCs to show localization of STX3 within the cells.

**Figure 8:**
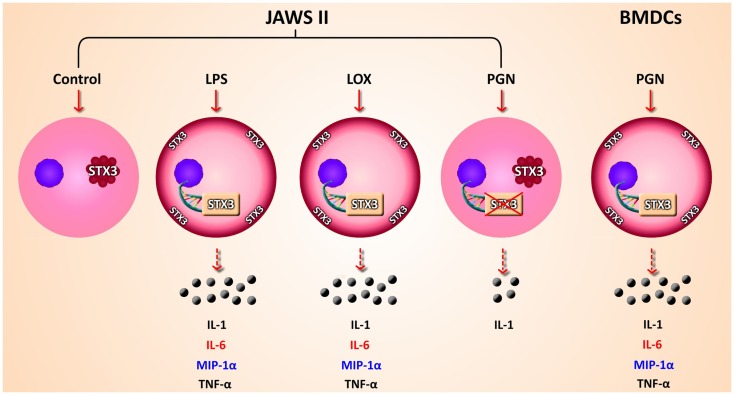
**Overview of findings**. Schematic representation of secretion of cytokines and chemokines secreted from JAWS II DCs and BMDCs in response to TLR activation. mRNA expression up-regulation and translocation of STX3 in DCs only secreting IL-6 (red) while MIP-1α (blue) was also secreted in these cells, not to the same level as in BMDCs

Given that we had shown correlation between secretion of IL-6 and increased expression of STX3 in both JAWS II DCs and BMDCs we next wanted to confirm this relationship by examining the expression of STX3 in IL-6-secreting and non-secreting DCs using confocal microscopy. STX3 was detected in the cytoplasm of control JAWS II DCs and following stimulation with LPS and Loxoribine STX3 translocated to the plasma membrane after 1 h. PGN stimulation in JAWS II DCs did not result in translocation of STX3 to the plasma membrane. In BMDCs, which do respond to PGN and secrete IL-6, activation with PGN resulted in STX3 translocation (Figure 7). Unstained cells and cells stained with primary STX3 antibody (1°) alone were visualized for auto-fluorescence while secondary antibodies alone were imaged for non-specific binding in every experiment (Figure S3 in Supplementary Material). An overview of our finding on STX3 in BMDCs and JAWS II DCs are summarized in Figure 8.

## Discussion

Several studies have addressed the role of SNAREs in cytokine release from immune cells, however, little is still known about the specific SNAREs involved in cytokine or chemokine secretion by DCs. Indeed, expression of SNAREs in immune cells, especially at the mRNA level has also not been well characterized. Investigating their expression in immune cells, such as DCs may provide valuable insights into their involvement in cytokine and/or chemokine secretion thereby uncovering potential new targets in inflammatory disease. Previous studies have used the correlation between SNARE expression and cytokine secretion as a way of elucidating candidate SNAREs involved in the secretion of these immune mediators (13). We took a similar approach and used the JAWS II DC cell line to profile the expression of SNAREs and subsequent secretion of cytokines and chemokines in DCs following exposure to ligands for TLR2, TLR4, and TLR7. We demonstrate that activation of DCs with these TLR ligands results in differential expression of SNAREs and subsequent secretion of cytokines and chemokines and describe a role for STX3 in secretion of IL-6.

Here, we have shown for the first time, the expression of SNARE proteins in DCs following activation with a range of TLR ligands. Interestingly, many of the SNAREs we examined did not change in expression over the 12-h time points we assessed. This was of interest as some SNAREs have been well reported to play a role in other immune cells. For example, an up-regulation of STX6 mRNA 2 h post stimulation with LPS has been reported in RAW 264.7 macrophage cells (3) and SNAP23 mRNA from the skeletal muscle of patients with type II diabetes was up-regulated (14). As SNAREs are used up during the fusion reactions and are recycled for numerous rounds of transport (15), we propose that along with caspases, other post-transcriptional regulation and recycling of SNAREs may be another reason for the lack of changes of SNARE mRNA expression in DCs.

We also did not detect any change in expression of VAMP8, which is one of the only SNAREs, which has been assigned a functionality in DCs to date. It has been previously indicated to be involved in phagocytosis in DC. Ho et al. reported that an over-expression of VAMP8 significantly inhibited phagocytosis suggesting that VAMP8 negatively regulates this process (11). The same group in another study notes that although they have indicated a role for VAMP8 in DCs, VAMP8 mRNA was not significantly elevated during DC maturation (16). This correlates with our data and Ho et al. accredited this to regulation at the mRNA level of VAMP8 by caspases (16). This may be indicative for a role for caspases and other molecules involved in post-transcriptional regulation of SNARE mRNA in DCs as VAMP8 mRNA has been shown to be up-regulated in other cells types such as over-reactive human platelets. Indeed hyper-reactive platelets have a higher fold expression of 4.8 compared to hypo-reactive platelets (17).

Our data does demonstrate significant up-regulation of SNAREs such as STX3, STX11, Vti1a, and Vti1b in DCs following activation. There was a significant up-regulation of Vti1a mRNA at 4 and 12 h post LPS and Loxoribine stimulation. It has previously been reported that up-regulation of Vti1a and Vti1b inhibits phagocytosis in DCs (10). As mature DCs are known to have reduced phagocytic ability (18) it may suggest that Vti1a was up-regulated to inhibit phagocytosis as the DC matured. There was also significant up-regulation of STX11 mRNA expression in response to LPS Loxoribine and PGN compared to control cells. STX11 is highly expressed in cells of the immune system and interestingly has an already established role in immune disease. Loss or mutation of the STX11 gene results in an autosomal recessive disorder known as, familial hemophagocytic lymphohistiocytosis type-4 (FHL-4), which causes immune dysregulation. This disorder is characterized by high levels of inflammatory cytokines and defective function of T cells and natural killer (NK) cells (19). STX11 has been reported to be up-regulated in DCs following LPS activation but *Stx11* deficiency did not appear to affect DC function (20). STX11 has also been indicated to regulate other cells of the immune system such as NK, CD8^+^ T cells, macrophage, platelets, and in human blood neutrophils where up-regulation of STX11 mRNA expression during differentiation has been reported (19–21). While it is clear from our study that STX11 regulation in DCs may be important we did not find a definitive correlation for this SNARE with the profile of cytokines or chemokines being secreted by the DCs.

One of most significant increases in mRNA expression over time was that of STX3. Expression of STX3 mRNA in JAWS II DCs significantly increased following activation with LPS and Loxoribine 1 and 4 h post stimulation and conversely was down-regulated following PGN activation. A role for STX3 has been documented in mast cells and epithelial cells but not yet in DCs. STX3 and VAMP7 have been shown to be important for apical transport of trans-membrane and secretory proteins in epithelial cells (22). Interestingly, a role for STX3 has been recently documented in the secretion of chemokines from mast cells. This study demonstrated that blocking STX3 activity with neutralizing antibodies inhibited the secretion of chemokines following IgE stimulation. The chemokines inhibited included IL-8, MCP-1, MIP-1α, and MIP-1β (12). As the JAWS II DCs secreted large amounts of MIP-1α it suggested that STX3 also has a role in chemokine secretion in DCs.

To elucidate a role for these up-regulated SNAREs we assessed the secretion of IL-6, TNF-α, and MIP-1α from JAWS II DCs following activation with TLR4, TLR7, and TLR2 ligands over the same time-course that the SNARE mRNA was profiled. IL-6 and MIP-1α were significantly secreted when JAWS II DCs were stimulated with LPS and Loxoribine but not with PGN. This was of particular interest to us as STX3 expression in JAWS II DCs was up-regulated significantly in response to LPS and Loxoribine but not to PGN stimulation. The concept of SNARE up-regulation paralleled with cytokine up-regulation has proven useful in other studies. Murray et al. correlated SNARE protein levels with TNF secretion from macrophage following LPS stimulation, which ultimately lead to the elucidation of STX4 functionality in macrophage secretion of TNF-α(3). The different roles of each SNARE in different immune cells is highlighted by the fact that in contrast to the role of STX4 in macrophage, we demonstrate a key role for this SNARE in secretion of IgE in B cells (23). With these factors in mind we examined STX3 in further detail to elucidate its functional roles in DCs.

We firstly depleted the levels of STX3 by means of a siRNA specific to STX3. In these experiments, IL-6 secretion was significantly down-regulated in the absence of STX3. MIP-1α was also down-regulated when STX3 was depleted by using RNAi. This correlates with recent data from mast cells where STX3 was reported to be involved in chemokine release from mast cells (12). Using confocal microscopy we assessed the cellular location of STX3 during activation with TLR ligands in both JAWS II DCs and BMDCs, and showed that translocation of STX3 only occurred in cells where IL-6 was secreted. This data indicates that STX3 has a role in IL-6 secretion (and possibly MIP-1α) from DCs and is an important SNARE in DC function.

IL-6 levels were not completely abolished from the JAWS II DCs and this may be explained as loss of SNAREs can be compensated by other members of the SNARE family, which have only 30% amino acid similarity (24). However, cytokines such as IL-6 are critical mediators of the autoimmune diseases and IL-6 is implicated in several disease processes. Increased levels of IL-6 have been observed in IBD, RA, systemic-onset juvenile chronic arthritis (JCA), osteoporosis, and psoriasis (25). IL-6 blockade has also been reported to be successful in autoimmune diseases and humanized anti-IL-6 receptor antibody is now routinely used in the clinic for the treatment of RA and JCA (26). In the past decade, IL-6 has been implicated with TGF-β in the differentiation of the Th17 subset, therefore its blockade may potentially improve these diseases at the pathogenic initiation phase (27).

The importance of future work in identifying SNARE complexes in immune function is highlighted by the award of a Nobel prize to Randy W. Schekman, Thomas C. Südhof and James E. Rothman who identified and functionally characterized these proteins and their involvement in trafficking. The potential to develop therapeutics that target SNAREs and suppress the secretion of cytokines may have significant advantages over therapies that target the effects of these cytokines post release. Indeed SNAREs may represent novel therapeutic targets in a wide range of disease states.

## Conflict of Interest Statement

The authors declare that the research was conducted in the absence of any commercial or financial relationships that could be construed as a potential conflict of interest.

## Supplementary Material

The Supplementary Material for this article can be found online at http://www.frontiersin.org/Journal/10.3389/fimmu.2014.00691/abstract
