# Supplementary figures and images for: A Role for Syntaxin 3 in the Secretion of IL-6 from Dendritic Cells Following Activation of Toll-Like Receptors
Source: Front Immunol. 2015 Jan 26;5:691. doi: 10.3389/fimmu.2014.00691 (PMC4306318; doi:10.3389/fimmu.2014.00691)

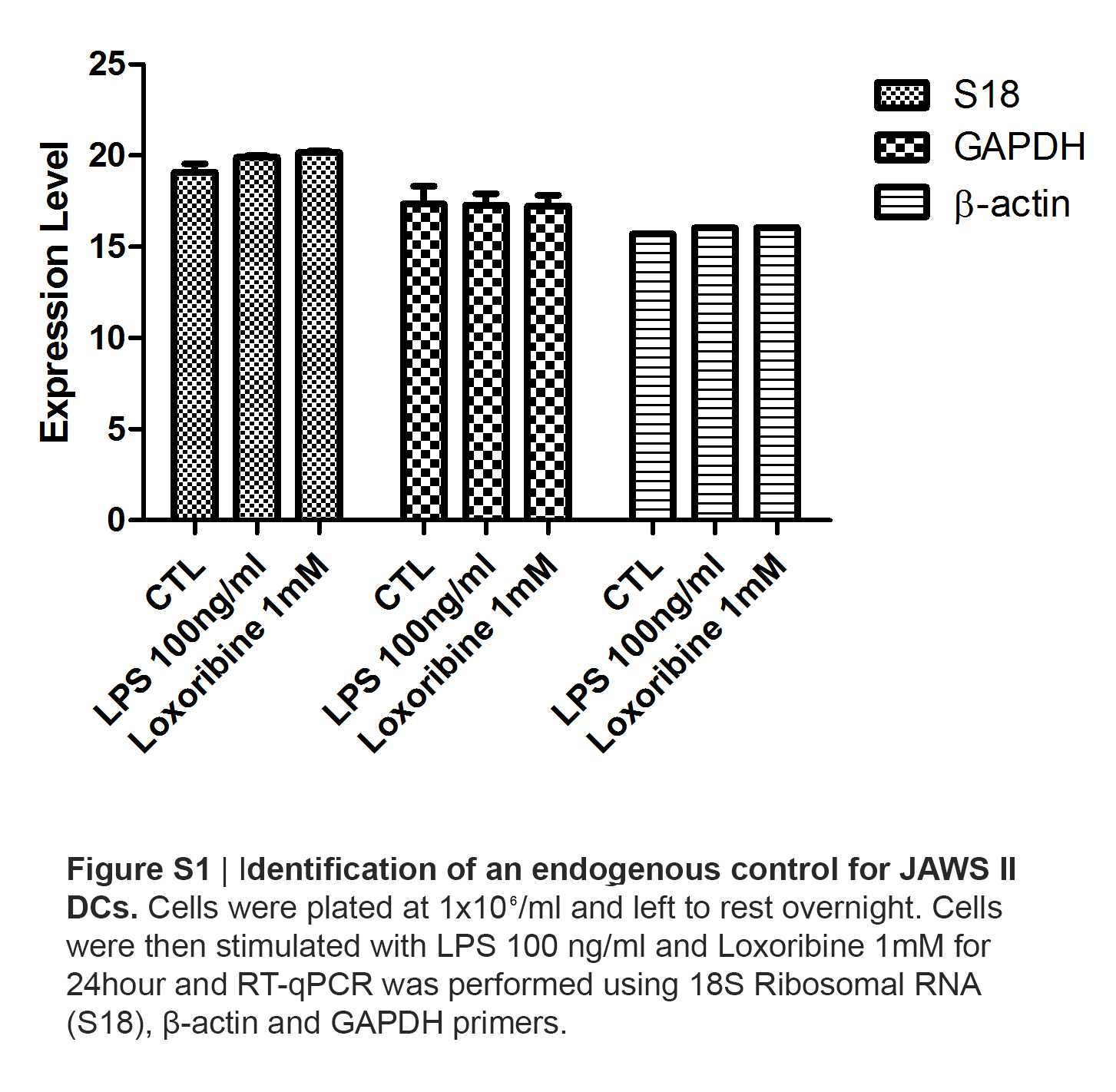

Supplement: Supplementary file 1 [file Image_1.JPEG]

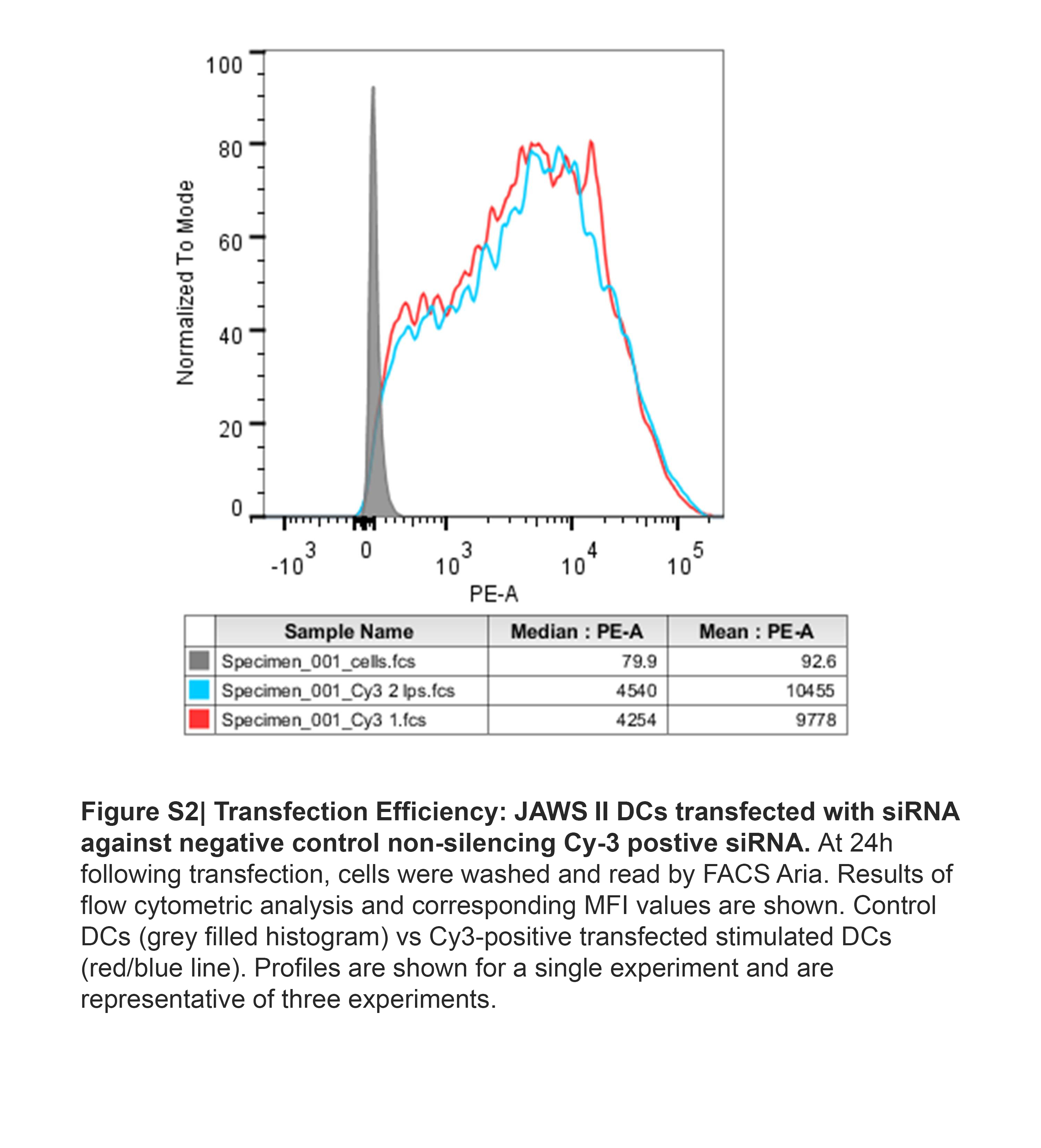

Supplement: Supplementary file 2 [file Image_2.JPEG]

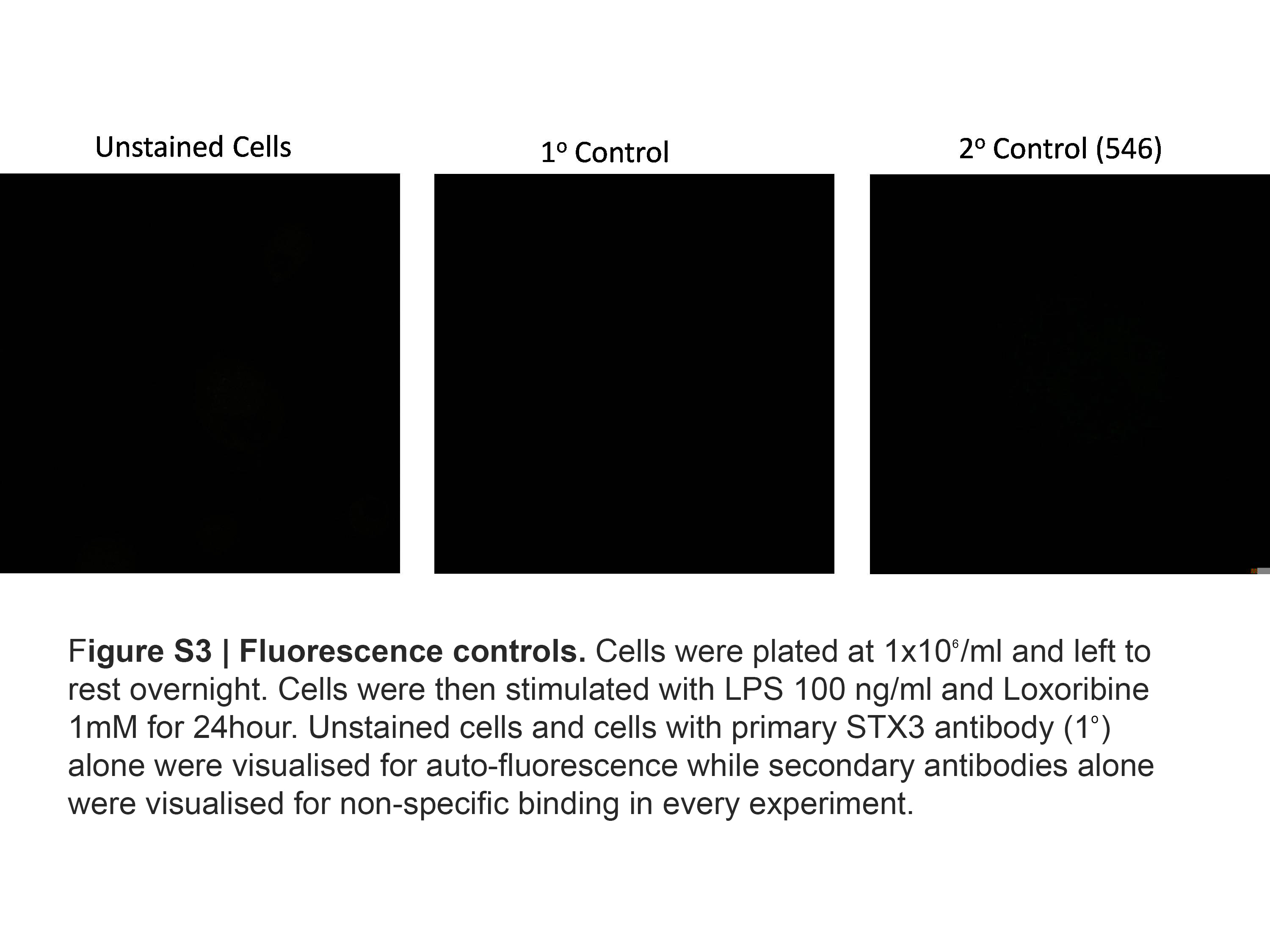

Supplement: Supplementary file 3 [file Image_3.JPEG]
